# Address

**Published:** 1872-06

**Authors:** H. A. Smith


### Dental Register.

VOL. XXVI.] JUNE, 1872. No. 6.]

### COMMUNICATIONS.

### ADDRESS

### Upon Retiring From the Presidency of the Mississippi Valley Dental Society, March 6th, 1872.

BY DR. H. A. SMITH.

*Gentlemen of the Association*: There is no subject perhaps more prominently before our profession at this time than *State Legislation in its relation to the practice of dentistry*. Nor one that is destined to affect a greater good for the profession as well as a real benefit to society at large, if the expectations of those who advocate this kind of legislation is in any degree realized.

The State of Ohio, as you know, was the first to enact a law compelling all persons who desired to practice dentistry in the State to give certain evidences of their fitness to pursue the calling. Since the passage of this act, in 1868, effort has been made, and is now being used with hope of success, to induce similar legislation in a number of the other States.

Dr Morgan, of Nashville, states that a dental law was in force in one of the Southern States—Alabama I think—more than thirty years.

It is my purpose on this occasion to direct attention to the more prominent features of this Ohio statute, that we may, as nearly as possible, understand its true spirit and object. To notice some of the objections that have from time to time been urged to it, and to give the results of some observation upon the practical working of the law, and the effect it has had upon the profession in the State.

Before proceeding to the main purpose before us, let me call attention to the fact that there has long existed a difference of opinion among those who have made the science of government a study as to the propriety of this class of legislation. In England at this time, the agitation of this question is being renewed in the discussion of what is termed the *Education Act*—a law, the object of which is the compulsory education of all the children of the State.

Prof. Huxley, an able advocate of this Education Act, has recently said, that this attempt to educate the people of England, "is one of the most hopeful events in modern history.

But strange as it may seem to the citizens of a country whose pride is a system of common school education, he adds that there is no inconsiderable number, "in whose judgment all this legislation is a step in the wrong direction, false in principle, and consequently sure to produce evil in practice." *Huxley's Address*—"*Administrative Nihilism.*"

This class of objectors would regard a law such as we we term a "Dental Law," or any act to regulate a trade or profession, as an unjust meddling with the natural rights of the individual. Recognizing as they do, that the duty of the State is embodied in the command, "Thou shalt not allow any man to interfere with the liberties of any other man," these objectors would class this with such as a compulsory vaccination law, an act to prevent the spread of a contageous disease, laws to prevent the adulteration of food or to prevent cruelty to dumb animals, and many others of a similar character. All these would alike come under their condemnation. Herbert Spencer, one of the ablest men opposed to this class of legislation, thus tersely states the principal that should be obobserved: "That until spontaneously fulfilled, a public want should not be fulfilled at all." And he further says, "To select out of an immense number of minor wants, physical, intellectual and moral, felt in different degrees by different classes, and by a total mass varying in every case, the want that is most pressing, is a task which no legislation can accomplish. No man or men, by inspecting society, can see what it most needs; society must be made to *feel* what it most needs. The mode of solution must be experimental, not theoretical. When left day after day to experience evils and dissatisfactions of various kinds, effecting them in various degrees, citizens gradually acquire repugnance to these proportionate to their greatness, and corresponding desire to get rid of them, which are likely to end in the worse inconvenience being first removed."—Essay—"*Over Legislation.*"

And this he claims "is a process far more trustworthy than any legislative judgments." Those who hold these views assert that the State rarely does any thing well beyond the prescribed sphere of guarding its citizens "against aggression, either individual or national." Not only do the laws of the State fail but they frequently make worse. Measures that promised reform only produced unlooked for evils. Mr. Spencer, in support of these views gives with many other examples of the failure of this class of legislation, this: "The examinations devised in England for insuring the efficiency of captains, (mercantile and marine), have had the effect of certifying the superficially-clever and unpracticed men, and rejecting many of the long-tried and most trustworthy: the general result being that the ratio of shipwrecks has increased." The same author in a recent paper says: "France has had for a century a state-established and a state-maintained school for educating skilled engineers. In England, until recently, no establishment of the kind has existed. The furnishing of engineering faculty was left to the law of supply and demand. The comparative results he asserts has been "railways originated in England not in France; that they spread through England faster than France, and that many railways in France were laid down by English engineers." This he gives as proof that "The spontaneous co-operation of men in pursuit of personal benefits will adequately work out the general good."

A little attention to the history of State legislation in our own country will discover a startling array of failures on the part of the law making power to prescribe wisely for the needs of society. That a good part of each session is spent in altering and repealing former laws, is conclusive that even the wisest legislators do not fully understand the laws of social science.

And the dental profession, who appear to be so largely in favor of legislative interference, should beware anticipating too soon, a realization of all the good they hope to themselves and the people from the most judicious State legislation to regulate the practice of dentistry.

Let us now turn our attention to the law, the first section of which I will read: "*Be it enacted by the General Assembly of the State of Ohio*, that it shall be unlawful for any person to practice dentistry in the State of Ohio for compensation, unless such person has received a dipioma from the faculty of a dental college, duly incorporated under the laws of this or any other State of the United States or foreign country, or a certificate of qualification issued by the State Dental Society, or by any local society auxiliary thereto; provided that nothing in this secton shall apply to persons now engaged in the practice of dentistry in the State before the first of January, 1873."

As the objections of opponents in nearly all cases embody a good deal of truth, I will first notice some of these. Early in the movement to obtain this legislation it was frequently asserted that the profession at large in the State did not want such a law. That the law was proposed and petitioned for by a mere faction who were influenced by certain educational interests. While it is true that in the start, as in every such effort, a few only of the members of the profession co-operated to procure the passage of the act—and among the number were some who were directly interested in the prosperity of a dental college—it is not true that they were influenced, except by a desire for the general good. The first suggestion that was acted upon came from Dr. C. H. James, at the time one of the younger members of the profession of this city. Dr. James although a graduate of a dental college, had no immediate interest in any such instiution. He was simply an earnest advocate of a thorough preparation and fitness of his profession for their calling; and no doubt every dentist in the State who engaged in securing this legislation was influenced by like motives.

Driven from this position the same objectors assert with more appearence of truth, that it is the dental profession only, and not the people of the State who want such legislation. The admission that the mass of citizens did not join in petitioning for it, is not conclusive that they would not have done so had they been solicited. On the contrary, whenever asked, citizens willingly joined in petitioning for such a law.

Neither is the fact that the movement did not orginate with the people, evidence that they did not desire or approve the action. The great mass of citizens do not as a rule, "ask spontaneously that a public want should be fulfilled." In instances of great imergenency, as when a contageous disease prevails,, calling for rigid sanitary measures, which the proper authorities fail to recognize, or there is carelessness or delay in enforcing a needed regulation of the kind, that the people take matters in their own hands and do what the officers of the State fail to do. If no law exists where one is much needed to prevent or correct an evil, and society at large fails to apply the needed remedy, in the way of State legislation: or as is probable the law-making power have no clear knowledge of the best way to reach and correct the evil, it would seem to be no interference with the rights of the citizen, for a profession or a body of men to propose and influence legislation which would be clearly for the good of the whole people. As well may this argument of the objector be applied to any movement looking to the correction of well-recognized evils, as to the "dental law." Take as a familiar example the society organized for the purpose of securing the needed legislation and enforcing the laws against the too common practice of cruelty to dumb animals. The movement though originating with a single person, cannot fail to receive the hearty approval of every humane citizen of the country. Every one recognized the existence of the evil before, but did not appreciate its enormity enough to move for its suppression. This labor was left to Mr. Berg and his associates. Now they have the united support of public opinion. And in this way a great reform is inaugurated, and the evil gradually disappears.

This legislative movement has discovered not a few in the dental profession, who appear to have a great respect if not an admiration for what they term "the old way." The former method of admission to the profession worked well—they say. Under it they assert—and with much truth—dentistry has advanced to its present proud position as a science; dental colleges have multiplied; dental associations, the great educators of the profession, have come rapidly into existence all over the country; and that many of the very men who advocate this reform, came into the profession under the old order of things, without the training of a dental college; that many of them are an honor to the calling and therefore they are a living refutation of that which they advocate. As it is well to weigh arguments against a reform, whether fair or unfair, let us see if there has been so great a departure from the "old way," as some would have us believe. Formerly all persons who desired, came into the profession without any restrictions. If they had the requisite knowledge they must have acquired it either at the professional schools, in the office of a preceptor, or by combining these advantages. Now the law does not, you observe, prescribe a new order of instruction. It does not say that you *shall* attend and graduate at a dental college. The person desiring to practice dentistry may, if he choose, disregard the law "that efficiency presupposes apprenticeship," and ignoring all the recognized methods of learning his profession, educate himself: thus adding his name to the long list of illustrious self-made men. There is no change in the methods of education. All that is required is that such person shall be a graduate of a dental college, or submit himself to an examination and show that he has, at least, some knowledge of the calling which he proposes to follow. A general education is not required; a professional education he must have. He must show that in some degree he is master of his craft. With less knowledge than this he is a "fraud, a snare." To protect the public against this deception is a principal purpose of the so-called reform.

It will be noticed that the law not only includes in its provisions those who seek to enter the profession, but those who were in actual practice of dentistry at the time of the passage of the act, and who had not a diploma from some dental college. There is no distinction as to privileges, only the latter class are allowed a fixed time, until 1873, to qualify themselves under the law. I would now ask your attention to this *retrospective* nature of the law, since it is to this feature that the most serious objection has been made.

A considerable number of the profession, myself among them, were apprehensive that if a law embodying this feature was passed, that it would not only arouse the opposition of those in practice who came under its provisions, but that when the time came for enforcing the law it would work serious injury to some who were dependent for a living for themselves and their families upon their earnings as dentists, but failing to pass examination would be compelled to seek other means of support, thereby subjecting them to great inconvenience and loss—if not positive suffering.

Although this may not be a valid argument against this particular provision of the law, especially if we accept the statement of Lock, that " The end of government is the good of mankind"—yet every humane man will naturally shrink from placing his fellow-man in a position whereby he may be made to suffer. The time has not come to fully test our fears in this direction. But since it has been my privilege to observe nearer the working of the law, I am free to say that the objections that I have mentioned are greatly magnified. The very fact that the greater proportion of those who might be inconvencied by this retrospective clause are the advocates and fast friends of the law, ought to relieve much of the apprehension that is felt in this direction. If they accept the situation, ought we to interpose objection?

In order, however, that a dental law which thus reaches back may not be oppressive, very much depends upon the discretion and good judgment of a board of examiners before whom this class of dentists are compelled to appear. Without a full appreciation of the responsibility which a decision in such cases imposes, no dentist would be qualified, in my judgment, to occupy a place upon such a board. I will relate an incident, of which some of us have knowledge, that may serve to illustrate what I have just said of this responsibility. A young man who had had but little opportunity to study his profession came into the State after the passage of this law, and began the practice of dentistry, thus bringing himself at once under its provisions. He presented himself before the State Board for examination. It was soon evident to him that he had not the requisite knowledge to intelligently practice his calling. He was permitted to withdraw, assuring the Board that he would apply himself industriously for a year in a course of study which was suggested to him, and then present himself for examination. If he then failed, he further stated, he would willingly abandon an occupation that he could not feel that he was fitted for, or legally authorized to follow, although by so doing he would sacrifice the only available means of providing for his young family and dependent parents. At the next annual meeting of the Board the plucky young dentist again appeared for examination. It was soon evident that he had been industrious in his studies, and made the best use of the time he had alllowed himself for preparation. The result was that he passed an examination that was alike creditable to himself and his profession. Had he failed and been compelled to abandon his occupation, with all these responsibilities I have named upon him—but this were best an unwritten history!

Lest seeming to give over much attention to these objections, I will now pass to notice some of the practical results of the passage of this "dental law."

The American citizen always applies this *test*, whether to individual action, a law, a principle, or even to a system of theology: "Is it a success?"

Anticipating the inquiry, is the law a success? I shall place myself at once on the defensive, and assume that it is too soon to put such a crucial test. It is not yet five years since the adoption of the law, and as it does not pass into full effect until the year 1873, it is not probable that a very marked change for the better should have manifested itself. I think it may be safely said, however, that the whole tendency of the effect produced by the law has been toward the improving and elevating the practice of dentistry in the State. Its influence may be noticed in an increased desire on the part of those persons about to enter the profession to more thoroughly prepare themselves in those departments of study which are now regarded as the only true basis of a sound dental education. Of the number who have passed the State Board of Examiners it may be said—though many of them did not need any special preparation—they have shown that they had been at work studying in those directions that an examination of the kind would indicate. The benefit of such study can not long be confined to the individual. His professional associates will be benefited, but more directly will his patrons reap the good. And of the profession generally throughout the State it may be remarked, that there is a spirit of inquiry abroad among them, which is shown in an increased interest in the State society, and a desire on the part of the members to investigate those subjects which underlie and lead to a correct practice, rather than giving that undue importance to merely practical or manipulative dentistry, which is so often a marked characteristic in our profession. The law has been effective in keeping out of the State some few, at least, of that class of so-called dentists who appear to have an inherent distaste for anything like a compulsory examination, lest it should discover to them their utter ignorance. While there is room and a welcome to the really efficient, our State is no longer a fit refuge for the incompetent dentist.

In conclusion, I think I may justly claim, without being influenced by an inordinate State pride, that the dental profession of the State of Ohio are justly entitled to a plaec in the front rank with their fellow-workers of the country.

While it is evident that our State dental law has had a beneficial effect, I would not forget in this estimate of the profession to give due credit to the influence which the Mississippi Valley Association has exerted in elevating the profession of the State to its present advanced position. Meeting here in the metropolis of the State year after year for a quarter of a century, you have given them a rare opportunity for improvement. As the more direct rays of the sun falling on the seed plant in the ground, causes it soonest to germinate and grow, so the profession of Ohio, being first subject to the light and knowledge which this association ever emits when any subject connected with dental science it brought before it could not fail to be warmed into a vigorous and progressive life.

Since our last annual meeting death has taken from amongst us a fellow member, Dr. J. G. Willis, of this city. He died May 23d. You remember him, the stal-worth, well balanced man, so full of life and energy and high hope, that he appeared one year ago. Of all our large membership he seemed least likely to succumb to the destroyer, death. Indeed, until within a few hours of his death, he stood manfully at his professional labors. Only two or three days before he was thus suddenly stricken down, it was my privilege to convey to Dr. Willis a request from the Trustees of the Ohio Dental College, that he accept a place as one of the corps of teachers in that institution. During this interview, he spoke particulary of his usual good health, and the regularity of his habits, lest he should in some way imperil it. He hesitated, because of not being quite willing to risk overtasking his strength to accept the position. "If I accept," he said, "I must do a good deal of hard work in the preparation, in order to do justice to the place and credit to myself, for I have enough of what may be called pride," he continued, "to do it well if I undertake to teach." This conversation shows how careful he was of his health, as well that quality or ambition of the man to always do his best; and it was this disposition that made him the careful practitioner that he was. He was not of the kind who looked upon the dental organs as so many inorganic bodies, to be operated on without regard to results. On the contrary, he noted every point that presented in any case, endeavoring to trace the relation of cause and effect. He was, therefore, a safe practitioner, one whose influence for good in the professsion was noticeably felt. His intercourse with the profession was marked by "thoughtfulness for others, generosity, modesty, and self respect." The qualities that make the gentleman.

While we involuntarily pay

> "The deep reverence taught of old,
> The homage of man's heart to death,"

let us not in our sorrow for the dead, forget to emulate the many excellencies of character of Dr. Wilis, both as a professional man and a citizen.

